# HBM4EU feasibility studies: Lessons learned in combining health and human biomonitoring studies: Hanna Tolonen

**DOI:** 10.1093/eurpub/ckac131.149

**Published:** 2022-10-25

**Authors:** HM Elonheimo, K Uusitalo, S Moore, A-M Andersson, K Wirkner, M Kolossa-Gehring, L Stewart, D Lermen, H Tolonen

**Affiliations:** 1 Finnish Institute for Health and Welfare, Helsinki, Finland; 2 University of Jyväskylä, Jyväskylä, Finland; 3 University of Eastern Finland, Kuopio, Finland; 4 Rigshospitalet, Copenhagen, Denmark; 5 LIFE, University of Leipzig, Leipzig, Germany; 6 University of Leipzig, Leipzig, Germany; 7 German Environment Agency, Berlin, Germany; 8 UK Health Security Agency, Oxfordshire, UK; 9 Fraunhofer Institute for Biomedical Engineerig, Sulzbach, Germany

## Abstract

**Background:**

The European Human Biomonitoring Initiative (HBM4EU) is a program protecting humans from the health effects of chemicals. The goal of HBM4EU is to make use of human biomonitoring (HBM) to assess human exposure to chemicals in Europe to better understand the associated health effects for citizens and to improve chemical risk assessment. Harmonisation and sustainable implementation of the HBM programme across Europe are important aims. In parallel to HBM studies, health examination surveys (HESs), dietary surveys, and disease specific health surveys are conducted in many European countries. In HESs, information collected by questionnaire(s) is supplemented with physical examinations and analysis of biomarkers from biological samples. HBM and HES use similar sample and data collection methods and infrastructures hence combining the two is being explored.

**Methods:**

Within HBM4EU, three feasibility studies (Finland, Germany, and UK/England) were conducted to evaluate opportunities and obstacles in combining HBM and health studies. We describe the contents and differences of these feasibility studies, and discuss the matters of shared benefits, obstacles, and lessons learned.

**Results:**

Benefits of combining HBM and HESs include the use of shared infrastructures, participants receiving additional health information from HES, and higher participation rates. Obstacles can be encountered when obtaining ethical approval and during time-consuming and complicated preparatory phases. Recruitment of participants and low participation rates are common concerns and designing participant-friendly questionnaires is important. Unexpected events such as the COVID-19 pandemic can cause challenges to studies. Furthermore, experiences from several countries demonstrated that long-term funding for combined studies can be difficult to obtain.

**Conclusions:**

In the future, incorporating HBM modules into HESs can provide a feasible and cost-effective method to conduct HBM studies.

**Key messages:**

• The European Human Biomonitoring Initiative (HBM4EU) protects humans from the health effects of chemicals in Europe. HBM4EU uses human biomonitoring (HBM) to evaluate human exposure to chemicals.

• In addition to HBM studies, health examination surveys (HESs) are conducted. In the future, incorporating HBM modules into HESs can provide a feasible and cost-effective method to conduct HBM studies.

